# Molecular Insights on Pathogenic Effects of Mutations Causing Phosphoglycerate Kinase Deficiency

**DOI:** 10.1371/journal.pone.0032065

**Published:** 2012-02-14

**Authors:** Laurent R. Chiarelli, Simone M. Morera, Paola Bianchi, Elisa Fermo, Alberto Zanella, Alessandro Galizzi, Giovanna Valentini

**Affiliations:** 1 Dipartimento di Biologia e Biotecnologie “L. Spallanzani”, Università degli Studi di Pavia, Pavia, Italy; 2 U.O. Ematologia 2, Fondazione IRCCS Cà Granda Ospedale Maggiore Policlinico, Milano, Italy; University Paris Diderot-Paris 7, France

## Abstract

Phosphoglycerate kinase (PGK) catalyzes an important ATP-generating step in glycolysis. PGK1 deficiency is an uncommon X-linked inherited disorder, generally characterized by various combinations of non-spherocytic hemolytic anemia, neurological dysfunctions, and myopathies. Patients rarely exhibit all three clinical features. To provide a molecular framework to the different pathological manifestations, all known mutations were reviewed and 16 mutant enzymes, obtained as recombinant forms, were functionally and structurally characterized. Most mutations heavily affect thermal stability and to a different extent catalytic efficiency, in line with the remarkably low PGK activity clinically observed in the patients. Mutations grossly impairing protein stability, but moderately affecting kinetic properties (p.I47N, p.L89P, p.C316R, p.S320N, and p.A354P) present the most homogeneous correlation with the clinical phenotype. Patients carrying these mutations display hemolytic anemia and neurological disorders, and,except for p.A354P variant, no myopaty. Variants highly perturbed in both catalytic efficiency (p.G158V, p.D164V, p.K191del, D285V, p.D315N, and p.T378P) and heat stability (all, but p.T378P) result to be mainly associated with myopathy alone. Finally, mutations faintly affecting molecular properties (p.R206P, p.E252A, p.I253T, p.V266M, and p.D268N) correlate with a wide spectrum of clinical symptoms. These are the first studies that correlate the clinical symptoms with the molecular properties of the mutant enzymes. All findings indicate that the different clinical manifestations associated with PGK1 deficiency chiefly depend on the distinctive type of perturbations caused by mutations in the *PGK1* gene, highlighting the need for determination of the molecular properties of PGK variants to assist in prognosis and genetic counseling. However, the clinical symptoms can not be understood only on the bases of molecular properties of the mutant enzyme. Different (environmental, metabolic, genetic and/or epigenetic) intervening factors can contribute toward the expression of PGK deficient clinical phenotypes.

## Introduction

Phosphoglycerate kinase (PGK) deficiency (OMIM 300653) is one of the relatively uncommon causes of hereditary non-spherocytic hemolytic anemia (HNSHA) which has gained the attention of physicians of different fields because a defective enzyme activity may also cause rhabdomyolysis, mental retardation, and various neurological disorders [1]. PGK (EC 2.7.2.3) is an essential enzyme for all living organisms. It catalyzes the reversible phosphotransfer reaction from 1,3-bisphosphoglycerate (1,3-BPG) to MgADP to produce 3-phosphoglycerate (3-PG) and MgATP, an important ATP-generating step in glycolysis. In addition to its physiological activity, human PGK can phosphorylate L-nucleoside analogues, which are used in antiviral and anticancer therapies [2]–[4]. Moreover, PGK was also shown to participate in the DNA replication and repair in mammal cell nuclei [5]. Finally, extracellular PGK has been recently reported to exhibit thiol reductase activity on plasmin, leading to angiostatin formation, which inhibits angiogenesis and tumor growth [6], [7].

PGK is a typical hinge-bending monomeric enzyme containing two nearly equal-sized domains that essentially correspond to the N- and C-terminal portion of the protein [8]. The N-terminal domain binds 3-PG or 1,3-BPG, whereas the C-terminal domain binds MgADP or MgATP. The two domains are separated by a deep cleft and linked by two alpha-helices (α-helix 7 and α-helix 14) [8], [9].

During the catalytic cycle the enzyme undergoes large conformational rearrangements, proceeding from an open form waiting for the substrates to a closed form performing the transfer of the phosphoryl group. Although conformational changes are promoted by substrate binding, only the concerted action of both substrates is able to trigger the domain closure, which leads to the proper geometry of the active site. Four hinge points contribute to the interdomain motions. Upon binding of the ligands the bending becomes restrained to a single hinge dominant point [9].

PGK requires magnesium ions for its activity and is characterized by an unusual kinetic behavior toward both substrates, being activated at high concentrations of either 3-PG or MgATP [10]. Thus, the kinetic profiles of PGK do not obey a simple Michaelis-Menten model and Lineweaver-Burk plots are biphasic. The enzyme is also activated by low concentrations of various multivalent anions, such as pyrophosphate, sulfate, phosphate, citrate. The anion activation, which is displayed at low substrate concentrations, seems to make the Lineweaver-Burk plots linear toward substrates [11]. The rationale of the kinetic behavior of PGK has not been so far unraveled, although basing on crystallographic studies an enzyme model has been suggested in which a secondary regulatory site is formed upon domain closure, in addition to the primary catalytic site [12]. Thus, at low concentrations the anion can bind to the regulatory site and increases PGK activity, whereas at high concentrations the anion can substitute the substrate at the catalytic site and therefore acquires inhibitory functions [8].

Two human phosphoglycerate kinase isoenzymes, PGK1 and PGK2, have been so far identified, characterized by distinctive tissue localization and encoded by two distinct genes [13], [14]. PGK1 is ubiquitously expressed in all somatic cells, including the red blood cells (RBC). Its gene maps to chromosome Xq13.3, spans approximately 23 kilobases and contains 11 exons and 10 introns [15]. PGK2, also known as testis form, is unique to meiotic/postmeiotic spermatogenic cells, and is expressed by an intronless gene which maps to chromosome 6p12–21.1 [14], [15]. The *PGK2* gene is a retroposon which arose by reverse transcriptase-mediated processing of a transcript from *PGK1* gene. In the human genome two non-functional pseudogenes have also been detected both presumably derived from the *PGK1* gene and mapping to chromosome Xq12 and 19p13, respectively [13].

PGK1 and PGK2 isoenzymes are structurally and functionally similar. They are both 417 amino acid-long with 87–88% amino acid sequence identity, and an apparent molecular mass of approximately 45 kDa.

PGK1 deficiency is inherited as an X-linked recessive trait. Thus, males have full expression of the disorder, whereas females are usually asymptomatic sharing a population of deficient cells coexisting with a normal cell population. Since the first description by Kraus et al. [16], nearly 40 patients with PGK1 deficiency have been reported, 27 of them characterized at molecular level.

Twenty different mutations have been so far identified (Table 1) [17]–[40]. Fifteen are missense mutations, two deletions of the coding region and three alterations of the splicing site. PGK1 deficiency is generally associated with moderate to severe non-spherocytic hemolytic anemia, often accompanied with central nervous system (CNS) disorders. In some cases PGK deficient patients exhibit muscular disorders. Mental retardation, behavioral abnormalities, seizures or strokes represent the main neurological alterations, whereas cramps and myoglobinuria characterize the myopathic forms. Interestingly, patients generally exhibit myopathy only after prolonged physical exercise. The reasons for the phenotypic variability associated with mutations of the *PGK1* gene are still unknown and worthy unraveling.

**Table 1:** *PGK1* mutations and clinical features in patients with PGK1 deficiency.

Nucleotide Change	Amino acid change	Variant name	n° of patients	age of diagnosis (years)	RBC PGK residual activity (%)	Muscle PGK residual activity (%)	Hb (g/dl)	Reticulocytes (%)	RBC 2,3-BPG increased	Symptoms			References
										A	M	N	
c.140 T>A	ap.I47N	Barcelona	1	3	8	N.A.	6.6–7.3	N.A.	**+**	**+**	**−**	**+**	[17]
c.266 T>C	ap.L89P	Matsue	1	9	5	N.A.	N.A.	N.A.	**+**	**+**	**−**	**+**	[18]
c.473 G>T	ap.G158V	Shizuoka	1	27	1	N.A.	12.8	2.5	**−**	**−**	**+**	**−**	[19]
c.491 A>T	ap.D164V	Amiens/New York	7	2–19	5	N.A.	2.0–10.0	5.0–26.0	**+**	**+**	**−**	**+**	[20]–[23]
c.571>573 delAAG	ap.K191del	Alabama	1	36	4	N.A.	14.1	6.4	N.A.	**−**	**−**	**−**	[24]
c.617 G>C	ap.R206P	Uppsala	1	26	10	N.A.	5.6–13.7	5.6–13.7	**+**	**+/−**	**−**	**+**	[25], [26]
c.755 A>C	ap.E252A	Antwerp	1	25	6	8	13.2	N.A.	N.A.	**−**	**+**	**−**	[27]
c.758 T>C	ap.I253T	Hamamatsu	1	11	8	4	N.A.	N.A.	N.A.	**−**	**+**	**+**	[28]
c.796 G>A; c.798 C>G	ap.V266M	Tokio	1	6	10	N.A.	9.3	12.5	**+**	**+**	**−**	**+**	[29]
c.802 G>A	ap.D268N	Munchen	population survey		21	N.A.	N.A.	0.4–1.3	**−**	**−**	**−**	**−**	[30]
c.854 A>T	ap.D285V	Herlev	1	68	49	N.A.	9–10	10–45	N.A.	**−**	**−**	**−**	[31]
c.943 G>A	ap.D315N	Creteil	1	31	3	5	14.3	N.A.	**+**	**−**	**+**	**−**	[20]
c.946 T>C	ap.C316R	Michigan	1	9	10	N.A.	7.5–13.0	1.5–5.0	N.A.	**+/−**	**−**	**+**	[32]
c.959 G>A	ap.S320N	Murcia	1	6	36	N.A.	7.6	9.0	N.A.	**+**	**−**	**+**	[17]
c.1060 G>C	ap.A354P	Kyoto	1	3	6	N.A.	4.9–9.0	24.0	N.A.	**+**	**+**	**+**	[33]
c.1132 A>C	ap.T378P	Afula	2	18, 25	2	1	13.4–14.5	N.A.	N.A.	**−**	**+**	**−**	[34], [35]
IVS4+1 G>T	splicing alteration	North Carolina	1	12	3	2	N.A.	2.7	N.A.	**−**	**+**	**+**	[36]
c.637>640 delGGCG	frameshift	Fukui	1	36	6	3	N.A.	N.A.	N.A.	**−**	**+**	**−**	[37]
c.639 C>T	splicing alteration	-	2	16, 21	5	3	N.A.	N.A.	N.A.	**−**	**+**	**−**	[38], [39]
IVS7+5 G>A	splicing alteration	Fukuroi	1	33	14	10	N.A.	N.A.	N.A.	**−**	**+**	**+**	[40]

A: anemia (+/−: compensated hemolytic anemia with occasional hemolytic crises); M: muscular disorders after physical exercises; N: neurological disorders; N.A.: not available;

a variants considered in this study.

In this study, the properties of 16 mutant enzymes obtained as recombinant forms were investigated and compared to those of the recombinant wild-type enzyme with the final aim to define the properties of the protein, and to correlate them with the pathological outcome.

## Materials and Methods

### Materials

Restriction enzymes and Taq polymerase were purchased from New England Biolabs. AMV reverse transciptase from Roche Diagnostics. Oligonucleotides were synthesized by Invitrogen. Quick Change XL Site-Directed Mutagenesis Kit was from Stratagene. ATP, 3-phosphoglycerate (3-PG), glyceraldehyde 3-phosphate dehydrogenase (GAPDH), NADH, isopropyl-β-D-thiogalactopyranoside (IPTG) were from Sigma-Aldrich. Other chemicals were reagent grade.

### Construction of expression vector encoding *PGK1*

To obtain the nucleotide sequence encoding *PGK1*, peripheral blood (10 ml) was collected from a normal subject after obtaining written informed consent and approval from the Institutional Human Research Committee of Fondazione IRCCS Cà Granda Ospedale Maggiore Policlinico of Milano. After blood collection, subject's name was replaced with codes to ensure anonymity. The procedures followed were in accordance with the Helsinki International ethical standards on human experimentation. Total RNA, obtained from leucocytes by Trizol method [41] was reverse transcribed, and the entire cDNA was amplified by polymerase chain reaction (PCR, 94°C, 30 sec; 58°C, 30 sec; 72°C, 120 sec; 35 cycles), using primers designed according to the reference sequence (NCBI Reference Sequence: NM_000291.3). The forward primer was 5′-TCGTTGACCGAATCACCGAC; the reverse primer was 5′-GTGCATTCTAGAGTGCATATATTT. The product was cloned into pCRII-TOPO vector (TA Cloning Kit; Invitrogen) and sequenced. A transition A>G was inadvertently generated at nucleotide 117 after the stop codon.

The insert of the recombinant pCRII-TOPO vector was PCR amplified (94°C, 20 sec; 52°C, 30 sec; 72°C, 90 sec; 5 cycles; 94°C, 20 sec; 60°C, 30 sec; 72°C, 90 sec; 25 cycles) using 5′-CCGTCTTCATATGTCGCTTTCTAACAAGCTGAC as forward primer, and 5′-CCGCTGGAGCTATTAAATATTGCTGAGAGCATCCACC as reverse primer, which included *Nde*I and *Xho*I sites, respectively. After digestion, the *PGK1* cDNA was inserted into *Nde*I/*Xho*I sites of pET-23b(+) expression vector (Novagen). The recombinant expression vector obtained was designed pMM1. The insert was checked by sequencing.

### Construction of expression vectors encoding mutant PGK1 enzymes

To obtain mutant enzymes, pMM1 was subjected to site-directed mutagenesis using Quick Change XL Site-directed Mutagenesis Kit (Stratagene) and sense and antisense mutagenic oligonucleotides. In all cases, but two, the oligonucleotides contained a single mutated base at the middle of their sequence. In the case of p.V266M, the oligonucleotides contained two mutated bases, whereas in the case of p.K191del, the oligonucleotides were without the codon for lysine (Table 2). The presence of the desired mutations and the absence of unwanted additional mutations were confirmed by sequencing the inserts.

**Table 2:** Sense and antisense oligonucleotides used for site directed mutagenesis.

Mutations		oligonucleotides
p.I47N	forward	5′-CTGCTGTCCCAAGCAACAAATTCTGCTTGGAC-3′
	reverse	5′-GTCCAAGCAGAATTTGTTGCTTGGGACAGCAG-3′
p.L89P	forward	5′-GAACTCAAATCTCTGCCGGGCAAGGATGTTC-3′
	reverse	5′-GAACATCCTTGCCCGGCAGAGATTTGAGTTC-3′
p.G158V	forward	5′-CTTCACTTTCCAAGCTAGTGGATGTCTATGTC-3′
	reverse	5′-GACATAGACATCCACTAGCTTGGAAAGTGAAG-3′
p.D164V	forward	5′-GATGTCTATGTCAATGTTGCTTTTGGCACTGC-3′
	reverse	5′-GCAGTGCCAAAAGCAACATTGACATAGACATC-3′
p.K191del	forward	5′-CTGGTGGGTTTTTGATGAAGGAGCTGAACTAC-3′
	reverse	5′-GTAGTTCAGCTCCTTCATCAAAAACCCACCAG-3′
p.R206P	forward	5′-GAGAGCCCAGAGCCACCCTTCCTGGCC-3′
	reverse	5′-GGCCAGGAAGGGTGGCTCTGGGCTCTC-3′
p.E252A	forward	5′-GTGCTCAACAACATGGCGATTGGCACTTCTC-3′
	reverse	5′-GAGAAGTGCCAATCGCCATGTTGTTGAGCAC-3′
p.I253T	forward	5′-CAACAACATGGAGACTGGCACTTCTCTGTTTG-3′
	reverse	5′-CAAACAGAGAAGTGCCAGTCTCCATGTTGTTG-3′
p.V266M	forward	5′-GGGAGCCAAGATTATGAAAGACCTAATGTCC-3′
	reverse	5′-GGACATTAGGTCTTTCATAATCTTGGCTCCC-3′
p.D268N	forward	5′-GCCAAGATTGTCAAAAACCTAATGTCCAAAGC-3′
	reverse	5′-GCTTTGGACATTAGGTTTTTGACAATCTTGGC-3′
p.D285V	forward	5′-GATTACCTTGCCTGTTGTCTTTGTCACTGCTG-3′
	reverse	5′-GTCGTCACTGTTTGACAACAGGCAAGGTAATC-3′
p.D315N	forward	5′-CTGGATGGGCTTGAACTGTGGTCCTGAAAG-3′
	reverse	5′-CTTTCAGGACCACAGTTCAAGCCCATCCAG-3′
p.C316R	forward	5′-GATGGGCTTGGACCGTGGTCCTGAAAG-3′
	reverse	5′-CTTTCAGGACCACGGTCCAAGCCCATC-3′
p.S320N	forward	5′-GACTGTGGTCCTGAAAACAGCAAGAAGTATGC-3′
	reverse	5′-GCATACTTCTTGCTGTTTTCAGGACCACAGTC-3′
p.A354P	forward	5′-CCGGGGAACCAAACCTCTCATGGATGAG-3′
	reverse	5′-CTCATCCATGAGAGGTTTGGTTCCCCGG-3′
p.T378P	forward	5′-GGAGACACTGCCCCTTGCTGTGCCAAATG-3′
	reverse	5′-CATTTGGCACAGCAAGGGGCAGTGTCTCC-3′

The underlined letters indicate the mutated bases.

### Expression and purification of PGKI enzymatic forms

Wild-type and mutant enzymes were expressed in *E.coli* BL21(DE3) pLysS cells transformed with the selected plasmids after a 5-hours induction with 0.5 mM isopropyl-β-D-thiogalactopyranoside. The induction temperature was 37°C for the following enzymes: wild-type, p.R206P, p.E252A, p.I253T, p.V266M, p.D268N. To express the remaining enzymes the induction temperature was lowered to 25°C.

To purify the enzymes, cells from one liter culture were collected by centrifugation, suspended in 50 ml Buffer A (20 mM Tris(tris(hydroxymethil)aminomethane)-HCl pH 8.0, 1 mM EDTA, 2 mM β-mercaptoethanol), sonicated, centrifuged and the supernatant, after an additional centrifugation at 150.000 g, was applied to a 27×4 cm DEAE-Sepharose FF (GE Healthcare) column equilibrated in buffer A. The enzyme activity was eluted as a sharp single peak using equilibration buffer. The enzyme obtained from one liter culture was approximately 90 mg in the case of the wild-type recombinant form. Mutant enzymes were generally expressed at lower level (Table 3). Protein quantification was determined according to Lowry et al. [42].

**Table 3:** Expression of recombinant PGK1 enzymes.

	Induction temperature	Expressed PGK^a^ (mg)	Specific activity of purified enzyme^b^ (U/mg)
wild-type	37°C	99.9	816.0
p.I47N	25°C	13.0	229.2
p.L89P	25°C	7.1	573.1
p.G158V	25°C	8.8	104.3
p.D164V	25°C	30.1	55.0
p.K191del	25°C	55.3	99.4
p.R206P	37°C	77.7	738.6
p.E252A	37°C	59.4	501.2
p.I253T	37°C	66.9	862.0
p.V266M	37°C	9.5	735.1
p.D268N	37°C	52.8	457.5
p.D285V	25°C	52.5	47.4
p.D315N	25°C	35.6	185.4
p.C316R	25°C	37.1	253.3
p.S320N	25°C	8.2	282.1
p.A354P	25°C	53.9	287.2
p.T378P	25°C	27.2	73.0

a Obtained dividing the total PGK activity of the free cell extract by the specific activity of the purified enzyme. Data are referred to 1 L of cell culture.

b Determined at 5 mM 3PG and 5 mM Mg-ATP.

### Molecular mass determination

To determine the molecular mass of the native enzyme, the purified PGK1 (100 µl, 0.1 mg/ml) was subjected to an analytical gel filtration on a Superose 12 HR 10/30 pre-packed column (GE-Healthcare) equilibrated in buffer A. For column calibration the following proteins were used: aldolase (120 kDa), albumin (67 kDa), ovoalbumin (45 kDa), chimotrysinogen (25 kDa) and ribonuclease (14 kDa).

### Enzyme activity assay

The enzyme activity of PGK1 was determined at 37°C, with 3-PG and MgATP as substrates, by GAPDH coupled spectrophometric assay according to the method recommended by the International Committee for Standardization in Hematology [43]. The standard reaction mixture contained 100 mM Tris pH 8.0, 0.5 mM EDTA, 2 mM MgCl_2_, 0.24 mM NADH, 40 µg GAPDH, 5 mM 3-PG, and 5 mM MgATP, in a final volume of 0.5 ml. The reaction was started by adding enzyme solution (0.1–0.5 µg). One unit is the amount of enzyme catalyzing the oxidation of 1 µmol NADH/min under the above conditions.

### Enzyme kinetic studies

Enzymatic activity was assayed at 37°C by using various concentrations of 3-PG and MgATP under conditions identical to those described above except for substrates. Kinetic parameters were determined as follows: for 3-PG at fixed concentration of 5 mM MgATP; for MgATP at fixed concentration of 5 mM 3-PG. In all cases the reaction was started by adding the enzyme (0.1–0.8 U/ml) and the enzyme activity was assayed at least at 10 different concentrations of substrate. All measurements were performed in triplicate by using a Jasco V-550 UV/VIS spectrophotometer (Jasco-Europe).

Kinetic parameters were calculated according to Szilagyi et al. [12], and using the Sigma Plot software (SPSS Inc).

*k*_cat_, or turnover number, is the number of catalytic events per second. *K*_m_ is the substrate concentration at which the reaction velocity is half maximal. *k*_cat_/*K*_m_ is a measure of how efficiently an enzyme converts substrate to product at subsaturating substrate concentrations.

### Thermal stability assays

The thermal stability was measured by incubating the enzyme (0.1–0.2 mg/ml) at given temperatures in buffer A. At intervals, samples were removed, chilled and assayed for enzyme activity. Relative activity was calculated as the percent of the enzyme activity before the incubation; t_1/2_ is the time required by the enzyme to lose 50% of its activity at a given temperature; T_50_ is the temperature at which the enzyme loses 50% of its activity in 10 min.

## Results

**Figure 1:**
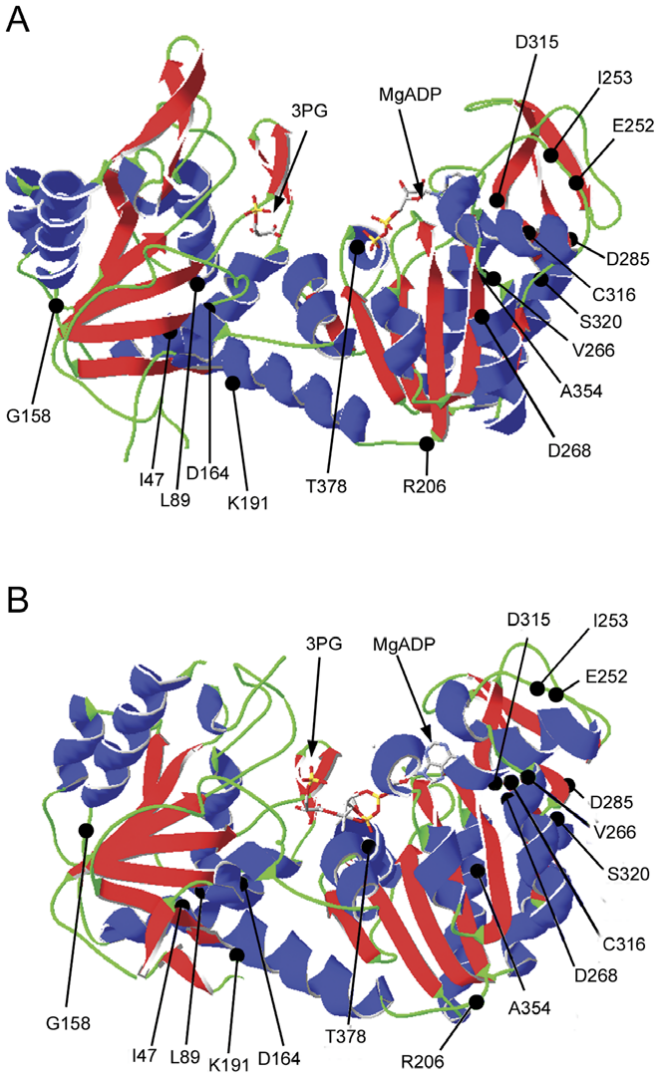
Ribbon representation of the human PGK1. Three-dimensional structure of open (A) and closed (B) human PGK1. The figures were generated from the atomic coordinates of Protein Data Bank, entry 2XE7 and 2WZC, using the Swiss-Pdb viewer (http://expasy.org/spdbv/). The black spheres indicate the Cα atoms of the amino acid residues subjected to mutagenesis. The arrows point to the substrates binding sites.

**Figure 2:**
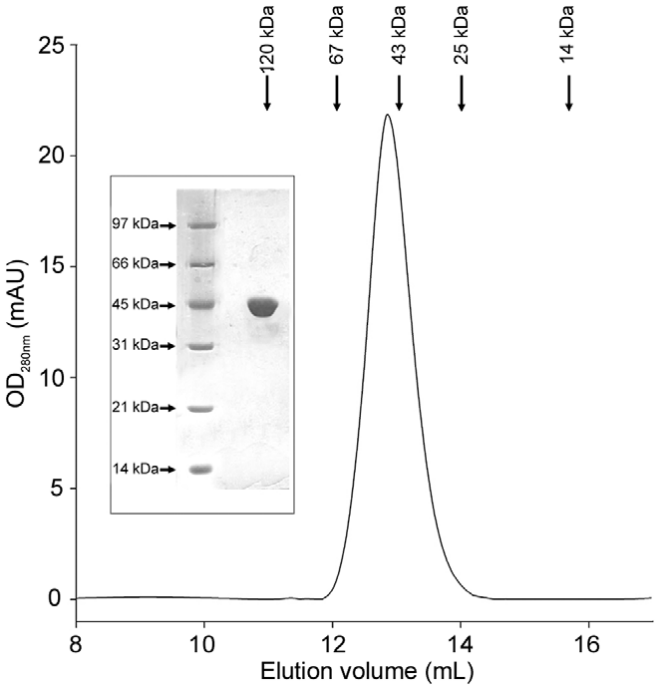
Assessment of the oligomeric state of recombinant PGK1. Elution profile of PGK1 from the analytical gel-filtration on a Superose 12HR 10/30 prepacked column. The position of the peak corresponds to a protein of approximately 45 kDa. The inset shows 12% SDS-PAGE of the purified PGK1 run in parallel with molecular mass standards, and stained with Coomassie Blue R-250.

To study the effects of genetic mutations on the molecular properties of PGK1, the desired modifications (Table 1) were introduced into the cloned cDNA of *PGK1* gene, and the mutated enzymatic forms were expressed in a bacterial system, purified to homogeneity and biochemically characterized. Maximal expression of most enzymes was obtained at 25°C (Table 3). The three-dimensional structure of the enzyme showing the amino acid residues affected by the mutations is represented in Figure 1. The adopted amino acid numbering is referred to the protein form which includes the initial methionine. Based on both 12% SDS-PAGE and analytical gel filtration chromatography, all enzymes examined were monomers of approximately 45 kDa (Figure 2).

### Protein thermal stability

**Figure 3:**
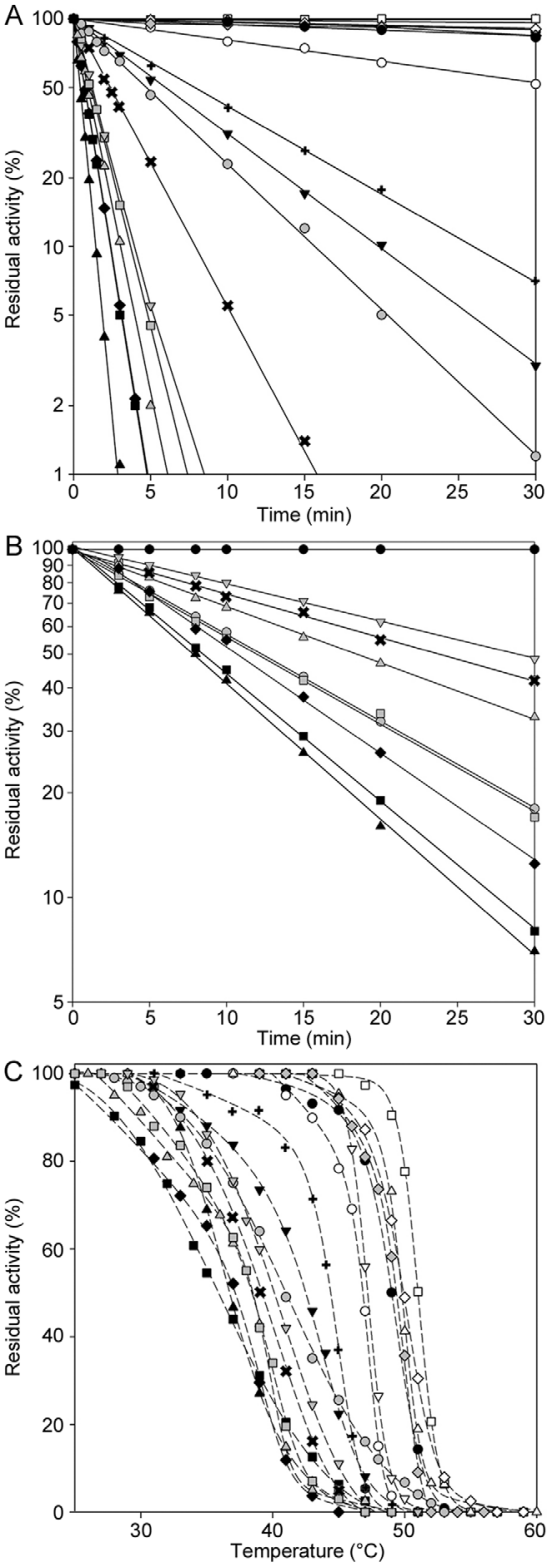
Thermal stability of PGK1 enzymes. Thermal stability of the PGK1 wild-type and variants at 45°C (panel A) and at 37°C (panel B). Each enzyme was incubated in buffer A and aliquots were collected at intervals for measuring residual activity. Plot of the residual activities at 10 minutes versus temperatures (panel C). Each enzyme was subjected to heat inactivation in a range of temperature from 25°C to 60°C. After 10 minutes of incubation at a given temperature, the enzyme sample was chilled and the residual activity measured. Residual activity was expressed as percentage of initial activity. •, wild-type; ▪, p.I47N; ▴, p.L89P; ▾, p.G158V; ⧫ p.D164V; 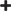, p.K191del; ○, p.R206P; □, p.E252A; ▵, p.I253T; ▿, p.V266M; ⋄, p.D268N; 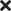, p.D285V; 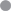, p.D315N; 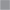, p.C316R; 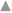, p.S320N; 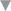, p.A354P; 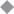, p.T378P.

Thermal stability was evaluated both in wild type and mutant PGK1 enzymes. All enzymes were initially treated at 45°C (the temperature commonly used in clinical analysis of PGK1 deficiency, Figure 3,A) and their half-life values (t_1/2_, see Material and Methods) were calculated (Table 4). The wild-type form was stable, retaining full activity after two hours of incubation at this temperature. One group of 5 mutant enzymes (p.E252A, p.I253T, p.V266M, p.D268N and p.T378P) had at 45°C a behavior similar to that of the wild-type enzyme (t_1/2_ values, >60 min) and, except for p.R206P, the remaining enzymes were highly sensitive to heat, halving their respective activities in less than 8 minute-incubation at 45°C. p.I47N, p.L89P, p.D164V, p.D285V, p.D315N, p.C316R, p.S320N and p.A354P showed the highest instability, their activities dropping to 50% after less than 30 minutes of incubation at the physiological temperature of 37°C (Figure 3,B).

**Table 4:** Thermal stability parameters of recombinant PGK1 enzymes.

	t_1/2_ 37°C (min)	t_1/2_ 45°C (min)	T_50_ (°C)
wild-type	stable	>60′	49.0
p.I47N	8′25″	0′43″	35.9
p.L89P	8′00″	0′25″	36.7
p.G158V	stable	5′54″	42.5
p.D164V	10′50″	0′43″	37.2
p.K191del	stable	7′48″	44.4
p.R206P	stable	32′00″	46.8
p.E252A	stable	>60′	51.0
p.I253T	stable	>60′	49.8
p.V266M	stable	>60′	47.2
p.D268N	stable	>60′	49.9
p.D285V	23′40″	2′24″	39.2
p.D315N	12′15″	4′42″	40.4
p.C316R	11′55″	0′56″	38.4
p.S320N	18′25″	1′13″	38.5
p.A354P	28′50″	1′12″	40.1
p.T378P	stable	>60′	49.4

Results are means (SE) for 3 determinations from at least 2 different protein preparations.

Moreover, an additional and more in-depth thermal analysis was performed incubating the enzymes at a wider range of temperatures (25°C–60°C; Figure 3,C), and calculating the temperatures at which they lost 50% of their respective activities after a period of ten minutes (T_50_). T_50_ was 49°C for the wild-type PGK1. Only 5 mutant enzymes (p.E252A, p.I253T, p.V266M, p.D268N, and p.T378P) had values comparable or even higher than that of the wild-type, all other variants tested had T_50_ values lower than the control. In some instances the T_50_ was reduced of more than 10°C (Table 4).

### Kinetic analysis of the wild-type PGK1 and mutant enzymes

The residual enzymatic activities reported in the literature associated with *PGK1* gene mutations are shown in Table 1. Enzyme activity is generally lower than 10% of normal, except for p.D268N, p.D285V and p.S320N variants (21%, 49% and 36%, respectively).

**Figure 4:**
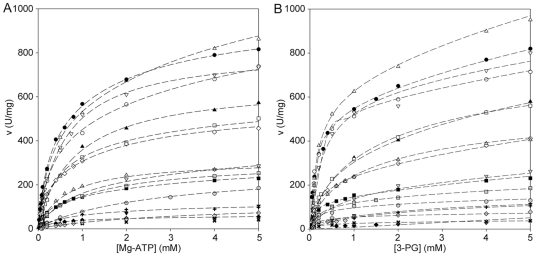
Steady state kinetics of PGK1 enzymes. Steady state kinetics of PGK1 wild-type and variants as a function of Mg-ATP at fixed 5 mM 3-PG (panel A) and as a function of 3-PG at fixed 5 mM MgATP (panel B). All experiments were performed at 37°C as reported in the "Material and Methods" section. •, wild-type; ▪, p.I47N; ▴, p.L89P; ▾, p.G158V; ⧫ p.D164V; 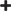, p.K191del; ○, p.R206P; □, p.E252A; ▵, p.I253T; ▿, p.V266M; ⋄, p.D268N; 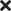, p.D285V; 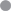, p.D315N; 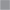, p.C316R; 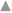, p.S320N; 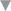, p.A354P; 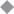, p.T378P.

In an attempt to understand the reason for the observed decreased activity, the kinetic properties of the wild-type and mutant enzymes were compared. Kinetic analyses were performed on the reverse reaction, at fixed concentration of free Mg^2+^ and using either MgATP (Figure 4,A) or 3-PG (Figure 4,B) as variable substrate. In all cases, the curve of velocity versus substrate concentration could not be fitted by a single hyperbola (non-Michaelian character), since the enzymes were activated by high concentrations of substrates. As a consequence the double reciprocal plots of the kinetic data were biphasic, represented by an interrupted straight line. For this reason the apparent *K*_m_ and *k*_cat_ values reported in Table 5 were obtained extrapolating the linear portion of the curves in the low substrate concentration range excluding the data affected by substrate activation. This range of substrate concentrations likely approaches the physiological situation.

**Table 5:** Apparent kinetic constants of recombinant wild-type PGK1 and mutant forms.

	Mg-ATP			3-PG		
	*k*_cat_ (s^−1^)	*K*_m_ (mM)	*k*_cat_/*K*_m_ (s^−1^ mM^−1^)	*k*_cat_ (s^−1^)	*K*_m_ (mM)	*k*_cat_/*K*_m_ (s^−1^ mM^−1^)
wild type	553.2±28.5	0.28±0.041	1975.7	468.2±33.3	0.17±0.011	2754.1
p.I47N	128.1±10.5	0.26±0.032	492.7	90.6±8.7	0.08±0.006	1132.5
p.L89P	249.4±33.7	0.23±0.070	1084.3	394.5±28.6	0.66±0.029	597.7
p.G158V	53.4±5.2	0.42±0.051	127.1	37.5±8.1	0.18±0.012	208.3
p.D164V	27.9±3.1	0.21±0.030	132.9	27.0±4.9	1.07±0.099	25.2
p.K191del	66.1±6.3	0.40±0.032	165.3	98.8±8.1	1.52±0.108	65.0
p.R206P	381.9±42.0	0.26±0.012	1468.8	450.1±29.6	0.15±0.020	3000.7
p.E252A	293.5±24.1	0.36±0.027	815.3	280.0±12.3	0.35±0.028	800.0
p.I253T	578.7±33.4	0.42±0.029	1377.8	588.0±31.0	0.27±0.014	2177.8
p.V266M	389.9±30.1	0.25±0.015	1559.6	499.1±24.5	0.29±0.011	1721.0
p.D268N	257.2±24.1	0.22±0.016	1169.1	215.1±10.7	0.26±0.018	827.3
p.D285V	24.7±2.2	0.27±0.018	91.5	27.2±1.9	0.34±0.020	80.0
p.D315N	134.7±9.1	1.36±0.060	99.0	79.1±5.7	0.12±0.009	659.2
p.C316R	98.7±6.3	0.21±0.009	470.0	116.1±8.4	0.30±0.011	387.0
p.S320N	104.1±5.7	0.19±0.010	547.9	257.1±11.4	0.47±0.022	547.0
p.A354P	225.9±12.4	0.87±0.080	259.7	201.0±9.1	0.53±0.025	379.2
p.T378P	44.9±3.3	1.43±0.065	31.4	47.1±3.4	0.15±0.010	314.0

Results are means (SE) for 3 determinations from at least 2 different protein preparations.

As for kinetics *versus* MgATP (Figure 4,A), the apparent *K*_m_ of the wild-type enzyme was valued at 0.28 mM, and the apparent *k*_cat_ at 553 sec^−1^. All variants showed an affinity toward this substrate similar to that of the wild-type, with the exception of p.D315N and p.T378P. In both cases, the apparent *K*_m_ value was five-fold higher than that of the wild-type enzyme (1.36 mM and 1.43 mM, respectively). For most mutants the catalytical rates were affected, although to a different extent. Outstanding are the apparent *k*_cat_ values of variants p.G158V, p.D164V, p.D285V and p.T378P that were 10% or less than that of the wild-type enzyme.

When 3-PG was the variable substrate (Figure 4,B), the wild-type enzyme exhibited an apparent *K*_m_ value equal to 0.17 mM and an apparent *k*_cat_ value of 468 sec^−1^. pD164V and p.K191del showed a significant reduction of their affinity toward 3-PG (apparent *K*_m_, 1.07 mM and 1.52 mM, respectively), whereas all other variants behaved like the wild-type enzyme. As far as the apparent *k*_cat_ was concerned, as in the case of MgATP, p.G158V, p.D164V, p.D285V and p.T378P showed values lowered to 10% that of wild-type enzyme.

## Discussion

The main objective of this research was to define how the enzyme alterations caused by PGK1 mutations could affect enzyme activity and generate the clinical manifestations of this disease. To this purpose, all the 20 mutations so far reported in literature were reviewed (Table 1) and 16 of them investigated at the protein level using purified preparations of the enzymes (Tables 4 and 5). The remaining four mutations were not considered, being predicted to have dramatic outcomes, such as complete absence of the protein product or an aberrant form of it.

The in-depth biochemical characterization of PGK1 variants shows that all mutations, with few exceptions, heavily impair the thermal stability and, to a different extent, the catalytic properties of the enzymes (Tables 4 and 5). Thus, the severity of molecular defects generally accounts for the remarkably low (mostly, <10% of normal) PGK activity observed in patients (Table 1).

A possible correlation with the different clinical manifestations of PGK deficient patients has been evidenced by grouping the characterized enzymatic variants according to their molecular defects (Table 6). The molecular interactions potentially affected by mutations, as inferred by the three-dimensional structures of human PGK1 in the open and in the closed form [44], [45], are shown in Table 7.

**Table 6:** Classification of mutants on the basis of their “in vitro” altered properties and the associated clinical phenotypes.

Mutation	amino acid affected by mutation		molecular impairments		symptoms		
	aconservation	blocalization	ccatalytic properties	dprotein stability	A	M	N
p.I47N	**++**	α-helix 1b	**+**	**+++**	**+**	**−**	**+**
p.L89P	**++**	α-helix 2	**+**	**+++**	**+**	**−**	**+**
p.C316R	**+**	β-strand q	**+**	**+++**	**+/−**	**−**	**+**
p.S320N	**+**	α-helix 11	**+**	**+++**	**+**	**−**	**+**
p.A354P	**−**	α-helix 12	**+**	**+++**	**+**	**+**	**+**
p.G158V	**+**	loop α-helix 4, β-strand E	**++**	**++**	**−**	**+**	**−**
p.D164V	**++**	β-strand E	**+++**	**+++**	**+**	**−**	**+**
p.K191del	**+**	α-helix 7	**++**	**++**	**−**	**−**	**−**
p.D285V	**++**	β-strand o	**++**	**+++**	**−**	**−**	**−**
p.D315N	**++**	β-strand q	**++**	**+++**	**−**	**+**	**−**
p.T378P	**+**	α-helix 13	**++**	**−**	**−**	**+**	**−**
p.R206P	**+**	loop α-helix 7, β-strand G	**−**	**+**	**+/−**	**−**	**+**
p.E252A	**−**	loop α-helix 9, α-helix 10	**−**	**−**	**−**	**+**	**−**
p.I253T	**++**	loop α-helix 9, α-helix 10	**−**	**−**	**−**	**+**	**+**
p.V266M	**+**	α-helix 10a/b	**−**	**−**	**+**	**−**	**+**
p.D268N	**+**	α-helix 10b	**−**	**−**	**−**	**−**	**−**

a ++: highly conserved; + conserved in vertebrates; − not conserved.

b according to Palmai et al. [9].

c catalytic efficiency toward 3-PG or MgATP: +++ <1%; ++ <10%; + <25%; − comparable to wild-type.

d heat stability (T_50_): +++ nearly 10°C lowered; ++ nearly 3–7°C lowered; + nearly 2°C lowered; − comparable to wild-type.

A: anemia (+/−: compensated hemolytic anemia with occasional hemolytic crises); M: muscular disorders after physical exercises; N: neurological disorders.

**Table 7:** Main interactions of the PGK1 amino acids involved in the mutations.

	open conformation^a^							closed conformation^b^						
	hydrogen/ionic interactions			hydrophobic interactions			solvent accessible^c^	hydrogen/ionic interactions			hydrophobic interactions			solvent accessible^c^
I47	N	→V44	O	CD1	→L60	CD2	no	N	→V44	O	CD1	→L60	CD2	no
	O	→C50	N	CG1	→L60	CD2		O	→C50	N	CG1	→L60	CD2	
	O	→L51	N	CG2	→L51	CD1		O	→L51	N	CG2	→L51	CD1	
				CG2	→L89	CD1					CG2	→L89	CD1	
L89	N	→L85	O	CD1	→I47	CG2	yes	N	→L85	O	CD1	→I47	CG2	yes
	N	→K86	O	CD1	→L85	CD1		N	→K86	O	CD1	→L85	CD1	
	O	→K91	N	CD2	→V44	CG1					CD2	→V44	CG1	
G158	N	→K156	O				no							no
D164	N	→R22	O				no	N	→R22	O				no
	O	→F166	N					O	→F166	N				
	OD1	→F188	N					OD1	→F188	N				
	OD1	→L189	N					OD1	→L189	N				
	OD1	→M190	N					OD1	→M190	N				
K191	N	→G187	O				yes	N	→G187	O				yes
	O	→N195	N					O	→N195	N				
								NZ	→D10	OD1				
R206	NH1	→K230	O				yes	NH1	→K230	O				yes
	NH2	→K230	O											
	NH2	→N232	OD1											
E252	O	→I307	N				yes	O	→I307	N				yes
I253	O	→S256	N	CD1	→F258	CB	no	O	→S256	N	CD1	→F258	CB	yes
				CD1	→F258	CG					CD1	→F258	CG	
				CD1	→F258	CD1					CD1	→F258	CD1	
				CG2	→F242	CB								
				CG2	→F242	CD1								
V266	O	→M270	N	CG1	→F244	CD1	no	O	→M270	N	CG1	→F244	CD1	no
				CG1	→V247	CB					CG1	→V247	CB	
				CG1	→V247	CG1					CG1	→V247	CG1	
				CG1	→L248	CD2					CG1	→L248	CD2	
				CG2	→F244	CD1					CG2	→F244	CD1	
				CG2	→F244	CD2					CG2	→F244	CD2	
				CG2	→F244	CE1					CG2	→F244	CE1	
				CG2	→F244	CE2					CG2	→F244	CE2	
				CG2	→F244	CZ					CG2	→F244	CZ	
D268	N	→I265	O				yes	N	→I265	O				yes
	O	→K272	N					O	→K272	N				
	O	→S271	N					O	→S271	N				
D285	N	→S320	OG				yes	N	→S320	OG				yes
	O	→G317	N					O	→G317	N				
	OD1	→G317	N					OD1	→G317	N				
	OD1	→S320	N					OD1	→S320	N				
	OD2	→E319	N					OD2	→E319	N				
D315	O	→V287	N				no	O	→V287	N				no
	OD2	→G351	N					OD2	→G351	N				
C316	SG	→S321	OG				no							no
S320	N	→D285	OD1				no	N	→D285	OD1				no
	O	→Y324	N					O	→Y324	N				
	OG	→V284	N					OG	→V284	N				
	OG	→D285	N					OG	→D285	N				
A354	N	→R350	O				yes	N	→R350	O				yes
								O	→E358	N				
T378	N	→G374	O				yes	N	→D375	O				yes
	O	→K382	N					O	→K382	N				
								OG1	→N36	ND2				

a Atomic coordinates of Protein Data Bank entry 2XE7;

b atomic coordinates of Protein Data Bank entry 2WZC;

c calculated with the CCP4 Suite, residues are considered solvent accessible when accessible surface area is >5 Å^2^.

One group of 5 mutant enzymes including p.I47N (Barcelona), p.L89P (Matsue), p.C316R (Michigan), p.S320N (Murcia), and p.A354P (Kyoto), are grossly perturbed in their protein stability and moderately affected in kinetic properties. A common clinical phenotype can be observed in patients carrying these mutations: all of them in fact display chronic hemolytic anemia and neurological dysfunctions, and except for p.A354P, no myopathy.

A second group of 6 variants comprising p.G158V (Shizuoka), p.D164V (New York/Amiens), p.K191del (Alabama), p.D285V (Herlev), p.D315N (Creteil), and p.T378P (Afula) display a great reduction of catalytic efficiency, mostly due to a cut of catalytic rate (*k*_cat_), and in some cases (p.D164V, p.K191del, p.D315N, and p.T378P) coupled with an increase of apparent *K*_m_ values (Table 5). Moreover, with the exception of p.T378P, all members of this group are highly heat sensitive, suggesting that the carriers of these mutations should suffer from a multisystem disease (erythrocyte, muscle, and CNS involvement). Intriguingly, with the exception of the carriers of p.D164V, all the other patients show neither chronic hemolytic anemia nor neurological dysfunctions. Three of them have myopathy.

Lastly, a group of 5 variants, namely p.R206P (Uppsala), p.E252A (Antwerp), p.I253T (Hamamatsu), p.V266M (Tokio), and p.D268N (Munchen) do not display heavy alterations of their molecular properties, on the whole behaving like the wild-type enzyme. The patients differ one from each other for clinical symptoms.

A more detailed discussion of each group follows.

The first group of mutations stands out from the others since it presents the most homogeneous correlation with tissues affected (erythrocytes and CNS). In addition, all patients were diagnosed in childhood (Table 1). The mutations affect above all the amino acid residues which play a main role in preserving protein structure. For instance, I47 and L89 are involved on both hydrogen/ionic and hydrophobic interactions in both open and closed conformations (Table 7). In addition L89 is substituted by an α-helix-destabilizing proline inside the α-helix 2. In a similar way the instability of the variant A354P can find an explanation in the introduction of the proline in α-helix 12. Therefore, a possible explanation for the absence of muscular dysfunctions is that the skeletal muscle has the capability to promptly replace the enzyme fraction damaged by a sudden increase of body temperature (fever or strenuous exercise gives rise to physiological temperature increase [46]). The mutant enzymes, although thermolable, have enough activity to allow sufficient metabolic flow in glycolysis.

Mature RBC face a different situation, being prevented from performing protein synthesis. Thus, an increased degradation rate of such variants leads to a decreased PGK1 content which primarily accounts for the enzyme deficiency and in turn for a reduced ATP production. Anyway, in these conditions RBC could count on the Rapoport-Luebering shunt for energy generation bypassing the PGK1 reaction [47]. Nevertheless, severe anemia is observed. It is conceivable, as previously suggested [48], that the true cause of the hemolysis is better ascribable to an increase of acidity or inhibition of several glycolytic enzymes (such as hexokinase, phosphofructo kinase, and pyruvate kinase) as a consequence of an increased intracellular concentration of 2,3-BPG, as already reported [17], [18], [20], [21], [26], [29].

As for the neurological dysfunctions, the tissue presumably does not promptly supply new enzyme to replace the damaged fraction, leading to a depletion of ATP. Moreover, the reduced production of 3-PG could affect the biosynthesis of some derived neuromodulators [49].

With the mutants of the second group, in which all amino acids affected by mutations are involved in hydrogen/ionic interactions (Table 7), we are faced by a tangled situation in which the occurrence of quite obscure factors has to be invoked from time to time to explain the reasons of the different clinical signs of patients. The carriers of p.G158V p.D315N and p.T378P are all characterized by muscle weakness and myoglobinuria after induced exercise, without chronic anemia and CNS dysfunction [20], [34], [35]. The present data highlight the deleterious effects of mutations on the enzyme (p.G158V, catalytic activity reduced to approximately 8%, T_50_, 6.5°C lowered; p.D315N and p.T378P, catalytic efficiency reduced to 5% and 1.5%, respectively, mainly as a consequence of lowered affinity toward Mg-ATP; T_50_ reduced by 9°C, only in the case of p.D315N). Peculiar is the behavior of the enzyme isolated from the RBC of patient with p.G158V substitution, that has characteristics similar to the wild-type [19]. A possible explanation is that the enzyme isolated from RBC is the ectopically expressed PGK2 isoenzyme. An ectopic expression of PGK2 can probably occur in those tissues where the activity of PGK1 is absent. This suggestion is supported by the fact that mutations leading to aberrant forms of PGK1 (the last 4 mutations in Table 1) are associated with a clinical phenotype similar to that of the carriers of p.G158V, p.D315N and p.T378P. The uncontrolled expression of PGK2 or epigenetic events have been already called into question to explain the mild phenotype associated to the in-frame deletion delAAG c.571>573 or delAAG c.574>576, which result in a PGK1 protein lacking one of the tandem lysine residues at position 191 or 192 of the polypeptide chain [50]. This mutation has been detected in a patient 36-year old, affected by PGK1 deficiency with episodes of hemolysis with jaundice, mainly in correspondence of febrile episodes [24]. The present data show that both lysine residues of this region are fundamental not only for preserving protein stability, as previously suggested [24], but most of all for ensuring 3-PG binding and catalytic function (Table 5). K191 and K192 are part of α-helix 7, a crucial region of the enzyme between the N-terminal and C-terminal domain. Deletion of one lysine is likely to shift the arrangement of the amino acid residues, affecting key interactions responsible for domain-domain communications and, although indirectly, for 3-PG binding (L189 with D164; E193 with H391, S393 and T394 of the hinge βL; F197 with F166 of α-helix 5 in the vicinity of 3-PG binding site) [8]. Mutation p.K191del should be deleterious for all tissues that primarily use glycolytic pathway to obtain ATP. Therefore, these tissues have to rely on the alternative PGK2 activity.

The carrier of p.D285V [31] was reported to be in good health along his life and the diagnosis of PGK1 deficiency was performed when he was elderly. Curiously, his RBC residual activity is 49% of normal, in strong contradiction with the serious enzyme alterations evidenced in this study. Conceivably, the p.D285V substitution abolishes the hydrogen interactions established by D285 with the backbone of G317, E319 and S320 in α-helix 11 (Table 7), thus affecting a H-bonding network essential for the molecule arrangement [31]. Noteworthy, the nucleotide substitution (c.854 A>T) was only observed in about 90% of the DNA studied and different hypotheses have been proposed to solve this riddle. The present data reinforce the suggestion that this mutation in the *PGK1* gene occurred postzygotically, with only a fraction of the cells in the soma carrying the mutation [31], thus leaving the carrier asymptomatic.

c.491 A>T mutation turns out to be the most deleterious at the protein level. In fact, p.D164V variant has a nearly 20-fold reduction in its catalytic rate toward both substrates and a six-fold reduction in its affinity for 3-PG, which is reflected in a substantial decrease in the catalytic efficiency toward this substrate (2-order of magnitude lower than that of wild-type). Moreover, the mutant enzyme is severely affected in its protein stability (t_1/2_ at 37°C, 11 min). Most probably, D164 is functionally important in both 3-PG binding, although indirectly, and in transmitting to C-domain the conformational changes induced by 3-PG binding. Besides, the molecular interactions engaged by D164 with its counterparts are crucial for preserving the native structure of the enzyme. D164 is located at the terminus of β-E strand in N-domain and interacts with the backbone of residues located at position 188, 189 and 190 of α-helix 7 of PGK1 (Table 7). c.491 A>T is the most frequent mutation affecting the *PGK1* gene, being found in 7 patients belonging to four different families [20]–[23]. All these patients have chronic hemolytic anemia and neurological dysfunctions, but no signs of muscular disorders.

With regard to the third group of mutations, data suggest that clinical manifestations of these patients are not the consequence of the amino acid substitutions in PGK1, the variants displaying features of the authentic enzyme, at least at physiological conditions.

p.D268N variant, described in a population survey [30], is associated with an asymptomatic clinical phenotype. The erythrocyte residual activity is 20% of normal (Table 1). Thus, as previously reported [51], the decreased enzyme activity, at least in the RBC, could be due to a decreased protein content ensuing from the nucleotide substitution.

The mutation causing p.E252A substitution is a nucleotide transversion (A>C) at nt 755 position of *PGK1* c.DNA, just adjacent to the 3′ end of exon 7. As a consequence, the consensus 5′ splicing sequence AGgt is changed to a nonconsensus sequence CGgt leading to a reduction of splicing efficiency (approximately only 10%) [27]. Thus, the highly reduced activity found in cells (8% in muscle, 6% in RBC) is most likely a result of a low content of PGK1, due to a reduced maturation of its mRNA. Thus, not surprisingly, the clinical phenotype (myophaty, but no anemia or neurological defects) is similar to that shown by the patients with other splicing mutations (Table 1).

The clinical manifestations associated to p.I253T, p.V266M and p.R206P mutations (recurrent myoglobinuria and mental retardation, but no hemolysis, in the first case [28]; hemolysis and neurological signs but no myopaty, in the second case [29]; mild hemolytic anemia and mental retardation, but no rabdomyolysis in the third case [25], [26]) can not be understood only on the bases of well-defined molecular properties of the mutant enzyme.

In conclusion, the hemolytic disorder associated with neurological dysfunctions is in general present in PGK deficient patients with variants unstable but only mildly affected in catalytic properties. Conversely, the myopathy without hemolytic or neurological symptoms is observed in some patients with variants heavily affected in both catalytic properties and protein stability (Table 6). Thus, different clinical phenotypes correlate with the distinctive type of perturbations caused by the mutations, stressing the need for the determination of the molecular properties of PGK variants to assist in prognosis and genetic counseling.

The occurrence of additional genetic and/or epigenetic factors that contribute to the phenotypic variability cannot be excluded. The ectopic expression of an isoenzyme has been already described, for instance, in the case of pyruvate kinase deficiency [52]. Pharmacological, nutritional and, more in general, environmental factors cannot be dismissed, especially in consideration of the paucity of reported cases of PGK1 deficiency. The secondary activities described for the enzyme, such as thiol reductase, replication and repair of DNA, may as well be responsible of some clinical manifestations.
